# Effects of “two-heart” nursing mode on the psychological state and quality of life of stroke patients

**DOI:** 10.1186/s12883-023-03439-5

**Published:** 2023-11-04

**Authors:** Feifei Zhou, Xumei Tao, Liping Wang, Bo Shen, Honglian Fang

**Affiliations:** Department of Geriatric Neurology, The Affiliated Brain Hospital of Nanjing Medical University, No. 264 Guangzhou Road, Gulou District, 210029 Nanjing, Jiangsu China

**Keywords:** Two-heart nursing model, Cerebral apoplexy, Cognitive function, Mental state, Quality of life

## Abstract

**Background:**

Evidence-based nursing (EBN) intervention is a nursing approach that uses credible scientific research findings as evidence, in conjunction with patient needs, to provide personalized nursing care tailored to the specific needs of patients. EBN has been widely applied in clinical practice and has achieved remarkable results. However, there are limited studies evaluating the efficacy of EBN on cognitive impairment, psychological disorders, and quality of life in stroke patients. This study aims to explore the clinical effects of the EBN, which we call “two-heart” nursing mode on cognitive function, limb function, mental state, and quality of life of stroke patients.

**Methods:**

A total of 92 stroke patients were divided into two groups: the traditional group (n = 46) and the two-heart group (n = 46). The traditional group received conventional nursing care, while the two-heart group received the double-heart nursing mode in addition to conventional nursing care. The cognitive function, limb function, living ability, mental state, quality of life, and nursing satisfaction of both groups were compared.

**Results:**

The cognitive function in the two-heart group (26.81 ± 3.15 points) was better than the traditional group (23.61 ± 3.74 points; P = 001); limb function in the two-heart group (86.16 ± 6.73 points) was improved compared to the traditional group (79.57 ± 5.19 points; P = 0.002), and the living ability of patients in the two-heart group (68.53 ± 5.87 points) was superior to the traditional group (60.79 ± 5.96 points; P = 0.003). Similarly, the quality of life of patients in the two-heart group (81.13 ± 6.69 points) was higher than the traditional group (70.78 ± 6.63 points; P = 0.003), and the mental state of patients in the two-heart group (43.61 ± 4.13 points, 43.19 ± 4.16 points) was better than that in the traditional group (50.59 ± 3.76 points, 51.49 ± 4.43 points; P = 0.003). However, the nursing satisfaction score in the two-heart group (97.83%) was slightly higher than the traditional group (95.65%; P = 0.068).

**Conclusions:**

The two-heart nursing mode can improve cognitive function, limb function, and mental state, as well as enhance the quality of life of stroke patients. This approach is worthy of clinical promotion and application.

## Background

Stroke, also known as a cerebrovascular accident, encompasses both ischemic stroke (cerebral infarction) and hemorrhagic stroke (including parenchymal hemorrhage, ventricular hemorrhage, and subarachnoid hemorrhage). It is a medical condition in which blood cannot reach the brain due to the rupture or blockage of blood vessels, resulting in brain tissue damage [1]. Currently, stroke is the leading cause of death among Chinese residents, with the incidence of ischemic stroke being much higher than that of hemorrhagic stroke [2]. The occurrence of stroke exhibits seasonality, peaking in winter and spring. The disease’s incidence is higher in the North compared to the South, higher in urban areas than rural ones, and more common in men than women. Additionally, there is an increased incidence of stroke in individuals with diabetes and obesity [3]. Due to the large population base and the aging population, the incidence of stroke is constantly increasing, and the number of patients is substantial.

Stroke can be caused by numerous factors such as hypertension, unhealthy lifestyle habits (e.g., smoking, staying up late), diabetes, hyperlipidemia, atrial fibrillation, obesity, overweight, hyperhomocysteinemia, transient ischemic attack, and severe internal carotid artery stenosis, among others [4–6]. Stroke onset is typically sudden, with early symptoms often going unnoticed. A small number of stroke patients may experience temporary limb weakness before the onset of the stroke. As the disease progresses, patients with ischemic stroke may exhibit weakness or numbness on one side of the limb, facial numbness or drooping, slurred speech or difficulty understanding language, blurred vision, difficulty seeing, altered consciousness, and difficulty walking [7, 8]. Hemorrhagic stroke patients may experience varying degrees of limb paralysis, altered consciousness, nausea, vomiting, and headache [9]. The condition significantly impacts the patient’s daily life and overall physical and mental health.

Stroke treatment options include general care, pharmacotherapy, surgical intervention, and traditional Chinese medicine [1, 10]. Regardless of the chosen treatment approach, proper and effective nursing care is essential to improve patients’ treatment adherence, encourage active cooperation with the treatment plan, and enhance the therapeutic effect. Evidence from prior studies has shown that specialized nursing care can help in reducing post-stroke depression and anxiety [11, 12]. The study conducted by Naess et al [12] revealed that nurse-led stroke aftercare, encompassing interventions such as psychoeducation, cognitive and emotional screening, and specialized care, demonstrated significant advantages in terms of emotional well-being when compared to standard care. Similarly, Zhang et al [11] provided evidence that a newly designed intensive caregiver education program, facilitated by nurses, can effectively mitigate anxiety and depression among patients diagnosed with acute ischemic stroke.

Evidence-based nursing (EBN) intervention refers to specialized nursing approach that incorporates scientifically sound research findings as evidence, in conjunction with patient requirements, to deliver individualized nursing care according to the specific needs of patients [13, 14]. EBN has gained significant attention in clinical settings and has been proven effective in the treatment and management of various medical conditions, including chest pain, postpartum depression, and acute coronary syndrome [15–17]. There is evidence suggesting that the utilization of EBN theories may lead to a reduction in the morbidity and mortality rates associated with stroke [14, 18]. However, there are limited studies evaluating the efficacy of EBN programs on cognitive impairment, psychological disorders, and quality of life in stroke patients. Therefore, in this study, we analyzed the nursing process and outcomes of 92 stroke patients admitted to our hospital and investigated the comprehensive impact of the Evidence-based nursing (EBN) intervention, which we called "two-heart" nursing model on the cognitive function, limb function, mental state, and quality of life in stroke patients.

## Methods

### Participants

We selected a total of 92 stroke patients admitted to our hospital from February 2021 to July 2022 following inclusion and exclusion criteria. The patients were divided into two groups: the Traditional Group (n = 46) and the Two-Heart Group (n = 46).

Inclusion criteria were: [1] stroke diagnosis confirmed by cranial CT, cranial magnetic resonance imaging, cerebral angiography, and differential diagnosis; [2] complete and accurate clinical data, informed consent, and voluntary participation; [3] acceptance of irregular follow-up.

Exclusion criteria included: [1] inability to continue participation or withdrawal; [2] hearing or vision impairments, disability of grade 3 or above [19], or communication difficulties; [3] mental illness or inability to communicate normally; [4] severe damage to vital organs; [5] presence of other severe diseases (e.g., neoplastic diseases, cardiovascular diseases, cerebrovascular diseases, renal failure, uremia, liver failure).

### Intervention

#### Traditional group: conventional nursing care

The Traditional Group received conventional nursing care. This included maintaining a clean and ventilated ward, explaining ward rules to patients and their families, and regularly changing the patients’ bedding. The patients’ conditions were monitored, and they were advised to follow the doctor’s medication instructions. In case of adverse reactions, the medical staff was informed to intervene and adjust treatment as needed. Health education, symptomatic nursing, and rehabilitation guidance were provided to enhance physical fitness and accelerate recovery.

#### Two-heart group: “two-heart” nursing mode

Patients in the Two-Heart Group received the "Two-Heart" EBN nursing method in addition to routine nursing. The evidence-based nursing procedure was as follows:

#### Questioning proposing

A. How to effectively improve the patient’s cognitive and limb functions after stroke.
B. How to implement effective psychological suggestion to reduce anxiety and depression for patients with stroke.
C. How to improve the patient’s post-stroke quality of life and nursing effect.

#### Evidence-based method

We retrieved relevant stroke nursing literatures by searching the Web of Science, PubMed, Wanfang database, Cochrane Evidence-based Medicine Network, and other databases for evidence from the research field. Stroke, anxiety, depression, limb and cognitive functions, quality of life, living ability etc were among the search terms. We consulted relevant data and excluded literatures including nursing methods lacking accuracy, non‐statistical treatment, and indicators without evaluation.

#### Evidence-based support

The same treatment protocol was implemented, which covers the following aspects.

#### Assist treatment

Nurses actively cooperated with doctors during treatment and rounds, recorded patient condition changes in detail, and observed recovery progress. They also educated patients and their families on potential adverse reactions, the importance of adherence to prescribed treatment plans, and the correct limb placement to prevent abnormal muscle tension according to individual patients need.

#### Active communication

Nurses established trust with patients and their families through effective communication using techniques such as speaking softly and showing kindness. They assessed patients’ psychological states, provided guidance, and offered support through various communication methods, including contrast, incentive, transfer, and confusion-solving techniques. Nurses also used praise to motivate patients and build their confidence in fighting the disease.

#### Improve environment

Nurses created a comfortable and quiet environment for patients by maintaining proper ward brightness, temperature, and humidity. They introduced patients to the surrounding environment and medical equipment, explaining their therapeutic purposes and precautions, to reduce anxiety and promote a sense of security.

#### Health education

Nurses provided tailored health education to patients and their families within 7 days after the patient’s hospitalization according to their needs while considering their educational levels and preferences. The education included the following subjects: First, the patients’ and their family members received instruction on the fundamentals of stroke, including risk, causes, symptoms, therapies, and managements of stroke; Second, family members were taught on how to support patients in managing stress, anxiety, depression, and other unsteady moods; Third, family members were educated on healthy diets and appropriate feeding methods in order to increase patients food appetite; and lastly the family members were taught necessary skills to help patients perform daily rehabilitation training. Nurses used various methods, such as videos, images, printed materials, and simple language, to improve patients’ understanding of their condition, treatment methods, prevention measures, nursing methods, and precautions. Nurses also patiently answered questions and corrected misconceptions.

#### Rehabilitation training

Rehabilitation training for patients included:

(A) Cognitive function training: Nurses instructed patients to identify people, places, and times; guided them in number training and reasoning skills; and provided training in item classification, language, and recall abilities.
(B) Limb function training: Nurses guided patients through a progressive exercise program, starting with simple in-bed exercises and progressing to balance training, alternating exercises, and eventually walking and stair climbing.
(C) Training of living abilities: Nurses trained patients to perform daily activities, such as washing, bathing, using the toilet, eating, and dressing.

#### Discharge intervention

After discharge, patients received telephone follow-ups every month up-to one year to monitor their condition, provide guidance on diet and exercise, and assess medication compliance. Every two months, patients were evaluated for disease progression, mental state, and quality of life, and their cognitive function, physical function, and life abilities were tested.

### Observation indicators

#### Cognitive function

The Montreal Cognitive Assessment Scale (MoCA) was used to evaluate cognitive function before and after care in eight areas: orientation, calculation, abstract thinking, visuospatial skills, language, memory, executive function, and attention and concentration. The scale consists of 11 items with a total score of 30 points. Cognitive impairment is indicated by a total score between 0 and 25 (0 ≤ total score < 26), while a normal cognitive function is indicated by a total score between 26 and 30 (26 ≤ total score ≤ 30).

#### Limb function

The Fugl-Meyer Motor Function Assessment Scale (FMAS) was used to evaluate patients’ limb function in both groups before and after nursing care. The scale consists of 50 assessment items, including 33 for upper limb function and 17 for lower limb function. Each item is scored as 0, 1, or 2, with a maximum score of 100 points. Limb dysfunction is categorized as severe (0 ≤ total score < 50), moderate (50 ≤ total score < 96), mild (96 ≤ total score < 100), or normal (total score = 100).

#### Activities of daily living (ADL)

The Modified Barthel Index (MBI) rating scale was used to evaluate patients’ ADL in both groups before and after nursing care. The scale includes 10 items (eating, bathing, grooming, dressing, bowel control, bladder control, toilet use, transfer, ambulation, and stair climbing) with a total score of 100. Levels of dependence are classified as extreme (total score < 40), moderate (41 ≤ total score < 60), mild (60 ≤ total score < 99), or none (total score = 100).

#### Quality of life

The Stroke-Specific Quality of Life Scale (SS-QOL) was used to evaluate the quality of life before and after care across 12 aspects: physical health, family role, language, mobility, emotion, personality, self-care, social role, thinking, upper limb function, vision, and work ability. The scale includes 49 items, each with five response options. Higher scores indicate a better patient quality of life.

#### Psychological state

The Self-Rating Anxiety Scale (SAS) and Self-Rating Depression Scale (SDS) were used to evaluate the mental state (anxiety and depression) of patients in both groups before and after nursing care. The SAS comprises 20 items with four response options, scored as 0, 1, 2, 3, and 4. The total score ranges from 0 to 80, with classifications of no anxiety (0 ≤ total score < 50), mild anxiety (50 ≤ total score < 60), moderate anxiety (60 ≤ total score < 70), and severe anxiety (total score ≥ 70). The SDS also includes 20 items with four response options, scored as 0, 1, 2, 3, and 4. The total score ranges from 0 to 80, with classifications of no depression (0 ≤ total score < 50), mild depression (50 ≤ total score < 60), moderate depression (60 ≤ total score < 70), and severe depression (total score ≥ 70).

#### Nursing satisfaction

The hospital-designed Nursing Satisfaction Survey was used to evaluate the satisfaction of both patient groups concerning nursing care in the following areas: service attitude, nursing knowledge, nursing skills, and nursing outcomes. The survey used a weighted average scoring system with a total score of 100 points. Satisfaction levels were classified as: satisfied (90 ≤ total score ≤ 100), somewhat satisfied (80 ≤ total score < 90), neutral (60 ≤ total score < 80), dissatisfied (0 < total score < 60), and very dissatisfied (total score = 0). Overall patient satisfaction was calculated as follows: (satisfied cases + somewhat satisfied cases)/total cases × 100%.

### Statistical analysis

We used SPSS 20.0 software for statistical analysis. Counting and measurement data were expressed as percentages and mean (± standard deviation), respectively. Chi-square (x²) and t-tests were performed, with a p-value of < 0.05 considered statistically significant.

## Results

### Baseline data of the participants

The traditional group comprised 26 males and 20 females, aged 49–76 years (mean: 59.73 ± 6.95), with varying education levels. The two-heart group included 24 males and 22 females, aged 47–79 years (mean: 60.48 ± 7.26), with diverse educational backgrounds. There were no significant differences in gender, age, and education between the two groups, ensuring comparability (P ˃ 0.05).

### Comparison of cognitive function

The MoCA scale was utilized to evaluate patients’ cognitive function in both groups. In the traditional group, MoCA scores were 20.19 ± 3.27 before care and 23.61 ± 3.74 after care. For the two-heart group, scores were 20.42 ± 3.19 before care and 26.81 ± 3.15 after care. There was no significant difference between the two groups before nursing care (P˃0.05). However, the two-heart group demonstrated significantly better cognitive performance than the traditional group after giving the nursing care (P < 0.05) (Table 1).

**Table 1:** Comparison of cognitive function between the two groups (${\bar x} \pm {\rm s}$, points).

Groups	Number of cases	MoCA	
		Before care	After care
Traditional group	46	20.19 ± 3.27	23.61 ± 3.74
Two-heart group	46	20.42 ± 3.19	26.81 ± 3.15
t		0.341	4.439
P		0.367	0.001

Note: MoCA: Montreal Cognitive Assessment Scale.

### Comparison of limb function

Patients’ limb function was evaluated using FMAS in both groups before and after nursing care. We did not see any significant difference between the traditional group and the two-heart group FMAS scores (67.86 ± 5.79 vs 67.43 ± 5.35) before care (P˃0.05). Interestingly, there was a prominent improvement in the limb function of two-heart group patients (86.16 ± 6.73) when compared to the traditional group patients (79.57 ± 5.19) after care. The limb function of patients in the two-heart group was better than that in the traditional group (P < 0.05) (Table 2).

**Table 2:** Comparison of limb functions in different groups (${\bar x} \pm {\rm s}$, points).

Groups	Number of cases	FMAS	
		Before care	After care
Traditional group	46	67.86 ± 5.79	79.57 ± 5.19
Two-heart group	46	67.43 ± 5.35	86.16 ± 6.73
t		0.370	5.259
P		0.356	0.002

Note: FMAS: Fugl-Meyer Motor Function Assessment Scale.

### Comparison of patients’ living ability

The MBI rating scale was used to evaluate patients’ activities of daily living (ADL) in both groups before and after nursing care. The MBI scores for the traditional group were 46.98 ± 4.85 before care and 60.79 ± 5.96 after care. For the two-heart group, the scores were 46.69 ± 4.48 before care and 68.53 ± 5.87 after care. There was no significant difference between the two groups before care (P˃0.05). However, the ADL of patients in the two-heart group was better than that in the traditional group (P < 0.05) (Table 3) after care.

**Table 3:** Comparison of daily life ability of different groups (${\bar x} \pm {\rm s}$, points).

Groups	Number of cases	MBI	
		Before care	After care
Traditional group	46	46.98 ± 4.85	60.79 ± 5.96
Two-heart group	46	46.69 ± 4.48	68.53 ± 5.87
t		0.298	6.275
P		0.383	0.003

Note: MBI: Modified Barthel Index scale.

### Comparison of patients’ quality of life

The SS-QOL scale was employed to assess the quality of life in both groups before and after care. When compared the SS-QOL scores of the traditional group and two-heart group before care, there was no obvious difference between the two groups (55.61 ± 3.48 vs 55.32 ± 3.39) (P˃0.05). Though we observed a significant difference in the SS-QOL scale score between the two groups (70.78 ± 6.63 vs 81.13 ± 6.69) after care. The quality of life for patients in the two-heart group was better than that in the traditional group (P < 0.05) (Table 4).

**Table 4:** Comparison of quality of life of patients in different groups (${\bar x} \pm {\rm s}$, points).

Groups	Number of cases	SS-QOL	
		Before care	After care
Traditional group	46	55.61 ± 3.48	70.78 ± 6.63
Two-heart group	46	55.32 ± 3.39	81.13 ± 6.69
t		0.405	7.453
P		0.343	0.003

Note: SS-QOL: Stroke-Specific Quality of Life.

### Comparison of patients’ mental states

The SAS and SDS scales were used to evaluate the mental state of patients in both groups before and after nursing care. There was no significant difference in the psychological state (anxiety and depression) of the patients between the two groups (P˃0.05; Table 5) before nursing care. The traditional group SAS and SDS scores were 60.69 ± 4.95 and 62.76 ± 4.86 while the two-heart group SAS and SDS scores were 60.53 ± 4.36 and 62.93 ± 5.12. However, a significant improvement was seen in both groups after the nursing care (P˂0.05). In the traditional group, the SAS score reduced to 50.59 ± 3.76 while in the two-heart group, the SAS score was 43.61 ± 4.13 after care. Similarly, after nursing care, the SDS score was 51.49 ± 4.43 in the traditional group and 43.19 ± 4.16 in the two-heart group. Most importantly, the anxiety and depression score of the patients in the two-heart group was much lessened when compared to the traditional group, and the difference was statistically significant (P < 0.05) (Table 5).

**Table 5:** Comparison of psychological states of different groups (${\bar x} \pm {\rm s}$, points).

Groups	Cases	SAS		SDS	
		Before care	After care	Before care	After care
Traditional group	46	60.69 ± 4.95	50.59 ± 3.76	62.76 ± 4.86	51.49 ± 4.43
Two-heart group	46	60.53 ± 4.36	43.61 ± 4.13	62.93 ± 5.12	43.19 ± 4.16
t		0.165	8.476	0.163	9.263
P		0.435	0.003	0.433	0.003

Note: SAS: Self-Rating Anxiety Scale; SDS: Self-Rating Depression Scale.

## Comparison of nursing satisfaction

Regarding patient satisfaction with nursing, there were 35 cases (76.09%) in the traditional group and 44 cases (95.65%) in the two-heart group that showed satisfaction. Similarly, there were 9 cases (19.57%) in the traditional group and 1 case (2.17%) in the two-heart group that displayed somewhat satisfaction. However, no significant difference was observed in the overall patient satisfaction with nursing between the two groups (P > 0.05), as shown in Table 6.

**Table 6:** Comparison of patient satisfaction with nursing in different groups [case (%)].

Groups	Cases	satisfied	Somewhat satisfied	Neutral	dissatisfied	Very dissatisfied	Overall satisfaction
Traditional group	46	35(76.09)	9(19.57)	1(2.17)	1(2.17)	0	44(95.65)
Two-heart group	46	44(95.65)	1(2.17)	0	1(2.17)	0	45(97.83)
x² value							8.425
P-value							0.068

## Discussion

Stroke is a disease characterized by brain tissue and cell necrosis, which can be relatively fatal and disabling [20]. Patients are prone to adverse mental states during treatment. Moreover, the disease often leads to cognitive dysfunction and impaired limb function, as well as a series of adverse reactions, causing patients to gradually lose their ability to live independently and resulting in a decline in their quality of life. This has a negative impact on their daily life and physical and mental health [21]. Therefore, in addition to effective treatment, appropriate nursing intervention measures should be selected to provide comprehensive care for patients, improve their cognitive function, physical function, and living ability, accurately understand their mental state, and provide psychological care to improve their quality of life [22, 23].

This study investigated the impact of the two-heart nursing mode on stroke patients’ cognitive function, limb function, mental state, and quality of life. The findings demonstrated that the two-heart nursing approach significantly improved patients’ cognitive performance, limb function, mental well-being, and quality of life compared to traditional nursing. By providing comprehensive care, attentive communication, targeted health education, and ongoing support, the two-heart nursing mode addresses both physical and psychological needs, making it a valuable method for promoting recovery and enhancing the overall well-being of stroke patients, which is consistent with the results of previous studies [24–26].

Traditional nursing does not pay enough attention to patients [27]. Basic nursing is only given to patients when they are admitted to the hospital, which can only meet part of their nursing needs without understanding their mental state and comprehensiveness. Consequently, the nursing effect and care is often not ideal [28]. The two-heart nursing approach not only provides basic nursing services for patients but also offers more high-quality and efficient nursing services from various aspects, focusing on both the physical health and mental well-being of patients. This nursing mode supports doctors in treating patients with more attentive care and strives to provide detailed nursing services in a timely manner. Medical staff proactively use appropriate skills to communicate with patients, understand their thoughts and feelings, identify negative emotions in their families, and provide timely relief [29]. Encouragement and comfort are also given, praising patients’ correct behavior and ideas to motivate them and enhance their confidence.

Efforts are made to create a suitable ward environment for patients, providing a comfortable and quiet space for treatment and rest. Explaining the purpose of the treatment instruments and the surrounding environment can alleviate patients’ fear, unease, and nervousness. Targeted health education, based on the patients’ actual situation, can maximize the effect of health education, and make it easier for them to master health knowledge, thereby improving their nursing compliance [30, 31]. Training patients in cognitive function, limb function, and living ability can help them recover more quickly, consolidate treatment effects, and improve their quality of life [32]. Hospital discharge does not signify the end of nursing care. Through telephone follow-ups and home visits, patients’ conditions can be effectively controlled, and dietary and exercise guidance can promote the stability of their condition. This “two-heart” nursing care approach also allows for accurate knowledge of patients’ mental states, quality of life, cognitive function, limb function, and living ability recovery [33].

## Conclusions

In conclusion, the two-heart nursing mode can improve the cognitive function and limb function of stroke patients, enhance their mental state, and improve their quality of life.

## Data Availability

The datasets generated and/or analyzed during the current study are available from the corresponding author upon reasonable request.

## Abbreviations

EBN: Evidence-based nursing
MoCA: Montreal Cognitive Assessment Scale
FMAS: Fugl-Meyer Motor Function Assessment Scale
MBI: Modified Barthel Index
SS-QOL: Stroke-Specific Quality of Life Scale
SAS: Self-Rating Anxiety Scale
SDS: Self-Rating Depression Scale
